# Localized Bearing Fault Analysis for Different Induction Machine Start-Up Modes via Vibration Time–Frequency Envelope Spectrum

**DOI:** 10.3390/s24216935

**Published:** 2024-10-29

**Authors:** Jose E. Ruiz-Sarrio, Jose A. Antonino-Daviu, Claudia Martis

**Affiliations:** 1 Instituto Tecnológico de la Energía (ITE), Universitat Politècnica de València (UPV), 46022 Valencia, Spain; joruisar@die.upv.es; 2 Department of Electrical Machines and Drives, Technical University of Cluj-Napoca, 400114 Cluj-Napoca, Romania; claudia.martis@emd.utcluj.ro

**Keywords:** AC machines, vibration, bearing, fault diagnosis

## Abstract

Bearings are the most vulnerable component in low-voltage induction motors from a maintenance standpoint. Vibration monitoring is the benchmark technique for identifying mechanical faults in rotating machinery, including the diagnosis of bearing defects. The study of different bearing fault phenomena under induction motor transient conditions offers interesting capabilities to enhance classic fault detection techniques. This study analyzes the low-frequency localized bearing fault signatures in both the inner and outer races during the start-up and steady-state operation of inverter-fed and line-started induction motors. For this aim, the classic vibration envelope spectrum technique is explored in the time–frequency domain by using a simple, resampling-free, Short Time Fourier Transform (STFT) and a band-pass filtering stage. The vibration data are acquired in the motor housing in the radial direction for different load points. In addition, two different localized defect sizes are considered to explore the influence of the defect width. The analysis of extracted low-frequency characteristic frequencies conducted in this study demonstrates the feasibility of detecting early-stage localized bearing defects in induction motors across various operating conditions and actuation modes.

## 1. Introduction

Induction motors are widely utilized in the industry due to their well-known advantages and their outstanding economic trade off. The maintenance of such equipment represents an economic burden, since only in North America, millions of electrical machines must undergo repairs every year [1]. In particular, the most critical constructive elements in low-voltage induction motors are the rolling bearings placed between the housing and the rotating motor shaft [2]. The early diagnosis of such components is crucial for reducing plant maintenance costs and preventing hazardous scenarios. Bearing fault diagnosis represents a prominent research field and has attracted a vast amount of research interest over the last years [3]. Additionally, the advent of machine learning and the availability of numerous open-source bearing fault datasets (e.g., Case Western Reserve University (CWRU) [4], Paderborn University dataset [5], etc.) have greatly increased the volume of research in this area [6,7]. Nevertheless, the sole data-based identification of bearing defects lacks the physical understanding of the failure mechanics, which hinders cross-domain failure identification. Therefore, research exploring the effects of various bearing defects across different applications and scenarios remains crucial in the field.

Vibration monitoring continues to be the most widespread methodology for rotating machinery diagnosis, including electrical machines. One of the main reasons is the high number of standards based on this physical magnitude [8]. On the other hand, vibration monitoring in electrical machines offers insights for both mechanical and electromagnetic fault signatures [9]. The main drawback of vibration monitoring is the need for external sensors (i.e., normally accelerometers) attached to accessible non-rotating parts and the influence of different mechanical transfer paths accentuating or attenuating potential fault signatures [10]. Other authors have explored alternative techniques for bearing diagnosis in the field of electrical machines by utilizing less-invasive methods exploiting electromagnetic signatures. Radial vibrations caused by bearing defects induce an air-gap variation that can be sensed in the stator current [11]. Some works exploiting current monitoring for bearing diagnosis can be found in [12,13]. In addition to the current monitoring, other authors have explored the utilization of stray-flux to diagnose bearings. The literature emphasizes the utilization of statistical indicators to identify localized bearing faults, with no evident fault signature in induction machines for the acquired stray-flux signals [14,15]. Despite its disadvantages, vibration monitoring remains the preferred methodology for diagnosing bearing faults in induction machines over electromagnetic-nature techniques due to the direct relation between the defect and the acquired signal [16].

Vibration signal processing in steady-state conditions is a broadly studied discipline [17]. The literature identifies various techniques for effectively diagnosing bearing faults, particularly in scenarios where the signals exhibit periodic and time-invariant characteristics [18]. Envelope spectrum analysis is considered as the baseline frequency–domain technique for identifying signal-modulating components. This is a simple and historically effective technique that successfully identifies bearing localized fault signatures in the low frequency range [19]. Other popular techniques for steady-state signal processing are the Discrete Wavelet Transform (DWT) [20], Empirical Mode Decomposition (EMD) [21], and cyclostationary tools [22,23], among others.

Operation at a constant speed for prolonged time is not common in all motoring scenarios, which hinders fault detection by using the classic signal processing approaches. For this reason, signal processing techniques under variable speed conditions have been gaining research attention over the last years [24]. Among the most widely utilized methods are those based on order tracking. These methods leverage the inherent periodicity of fault components relative to the rotating frequency [18]. Consequently, all components can be represented in both the frequency and angular domains, ensuring compliance under variable speed conditions. These resampling techniques provide successful results for localized bearing fault diagnosis, as demonstrated in works such as [25,26]. Nevertheless, the sampling or estimation of the instantaneous frequency is necessary to perform the transformation into the angular domain [27], which imposes some limitations in terms of hardware and computational burden. Resampling-free techniques overcome the limitations of order tracking but involve a necessary post-processing step [24]. Some of the traditional methods to analyze non-stationary signals include linear methods such as the Short Time Fourier Transform (STFT) [28,29] the Continuous Wavelet Transform (CWT) [30], and quadratic bi-linear methods such as Winger–Ville Distribution (WVD) [31]. Moreover, bearing fault diagnosis triggers the application of advanced time–frequency signal processing techniques such as various types of synchrosqueezing transformations [32,33,34], or Multiple Signal Classification (MUSIC) [35,36]. These advanced techniques offer increased resolution and enhanced energy concentration at a computational cost. The complexity and computational requirements of such transforms represent a limitation in many diagnosis scenarios where simpler methodologies provide an adequate solution, as demonstrated in [37].

In the field of induction machines, bearing diagnosis during different operating conditions, including start-up, is identified as a research gap by many authors [38,39]. In [40], the authors identify defect early detection and severity quantification under non-stationary regimes and the utilization of low-computational-burden processing techniques to ease technology implementation as future research directions. In a recent review [41], the authors also highlight a research gap related to transient analysis under different operating regimes. Moreover, induction machines can be line-started, soft-started, or inverter-fed, which impose different mechanical and electromagnetic conditions that may affect bearing fault identification. Very recently, several authors focused on the vibration transient start-up signature of induction motors to diagnose rotor dynamic defects such as misalignment and mass unbalances [42,43]. Few authors have explored the start-up signature to detect bearing faults. In [44], the authors utilize the CWT and feature extraction algorithms to identify different bearing defects. Other authors have explored the transient start-up current signal for the same purpose [45]. In [37], the authors detect characteristic bearing fault signatures for inverter-fed machines via vibrations acquired in the bearing housing. Thus, the detection of bearing defects under different start-up modes in induction motors represents a research gap in the field.

This work analyzes localized bearing race defects in induction motors under various starting modes using a resampling-free, straightforward time–frequency transformation. The utilized signal processing tool extends classic vibration signal envelope analysis through the STFT. This paper aims to generalize the detection of incipient bearing faults via vibration signals across different defect widths, excitation modes, and operating regimes in low-voltage induction motors. Moreover, the transient results are analyzed along with their steady-state counterparts. The vibration data are generated in a custom test bench where vibrations are acquired in the Drive End (DE) of the machine housing for different constant load points and two bearing defect widths. Two different induction motor starting modes (i.e., line-started and scalar-controlled inverter-fed) are implemented to elucidate the main differences between them. In addition, the obtained signature is compared with an existing open-source dataset including inverter-fed transient vibration signals acquired in the bearing surroundings. This paper is structured as follows. Section 2 presents the mechanics of localized bearing defects and the employed time–frequency transformation tool. Section 3 describes the test bench in which the vibration data are generated and acquired. Section 4 presents the main results of the analysis and compares the obtained signature with existing datasets. Finally, Section 5 discusses the main outcomes and limitations, and Section 6 concludes this work and defines future research steps on the topic.

## 2. Theoretical Background

This section aims to present the fault mechanics of localized bearing defects, thereby enhancing the reader’s understanding of the analysis. Moreover, the basics of the utilized signal processing pipeline are described in a comprehensive manner.

### 2.1. Localized Bearing Fault Mechanics

**Figure 1:**
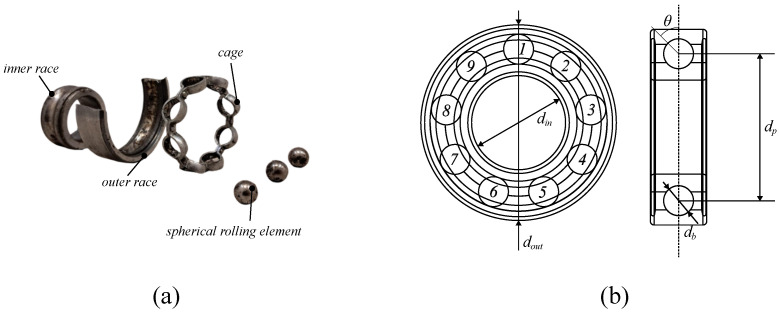
(**a**) Expanded deep-groove ball bearings view, (**b**) bearing geometry including numbering of rolling elements (i.e., 1 to 9 numbers) and main dimensions.

Single-row deep-groove ball bearings are extensively utilized in low-voltage electrical machines. These are formed by inner and outer races, rolling elements (in this case, spherical), and a cage equally spacing the rolling elements. Figure 1 shows an expanded view of a spherical rolling element bearing and the identifications of its main components. Other fundamental parts are the lubricants and the seals containing the lubricant.

Rolling bearings may suffer from a wide variety of faults of a different nature. These faults are typically categorized into three primary groups: localized, extended, and distributed defects. Distributed bearing defects are equally spaced over the bearing circumferential space. One classic example of distributed bearing fault is race fluting due to bearing currents [46]. On the other hand, localized and extended bearing defects are confined in space within the different elements of the rolling bearing (i.e., races or rolling elements). The main difference among these two defect types is the extension of the defect. The extended type may span a larger space than the localized counterpart. Some examples of localized defects are pits and cracks, while extended defects are commonly found in the form of fatigue spalling [47]. The present work focuses only on the localized race defect type, which is often recognized as the most incipient type of bearing fault.

Localized bearing faults are ideally understood as repetitive shocks caused by the contact between rolling elements and localized defects. These periodic signals are characterized by characteristic frequencies depending on the defect location and bearing geometry. In the ideal case of a fixed outer ring and a rotating inner race, these frequencies are defined as follows [48]:

(1) $$f_{BPO}=\frac{n_{b}}{2}\left[1-\frac{d_{b}cos\left(\theta \right)}{d_{p}}\right]f_{r}$$

(2) $$f_{BPI}=\frac{n_{b}}{2}\left[1+\frac{d_{b}cos\left(\theta \right)}{d_{p}}\right]f_{r}$$

(3) $$f_{C}=\frac{1}{2}\left[1-\frac{d_{b}cos\left(\theta \right)}{d_{p}}\right]f_{r}$$

(4) $$f_{BS}=\frac{d_{p}}{2d_{b}}\left[1-\frac{d_{b}^{2}cos^{2}\left(\theta \right)}{d_{p}^{2}}\right]f_{r}$$

where frequencies $f_{BPO}$, $f_{BPI}$, $f_{C}$, and $f_{BS}$ correspond to the ball pass frequency in the outer and inner raceways, the cage frequency, and the ball spin frequency, respectively. These are determined by the number of rolling elements ($n_{b}$), and geometric parameters, including the pitch diameter ($d_{p}$), ball diameter ($d_{b}$), and contact angle (θ), as shown in Figure 1b.

**Figure 2:**
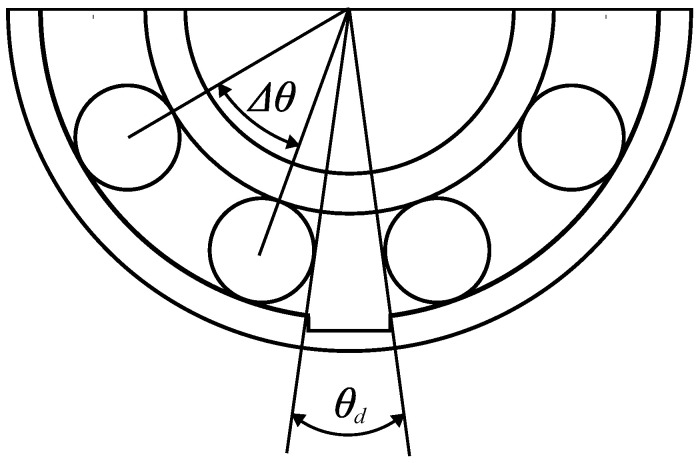
Defect ratio graphic description.

A more realistic scenario considers the existence of slippage between rolling elements and the races, which causes the defect impulses to adopt a quasi-periodic behavior [49]. In this case, the periodicity of the pulses experiences some random fluctuations, slightly affecting the characteristic fault frequency locus. The prediction of the exact amplitude of the overall vibration signal represents a complex mechanical problem including non-linear multi-body dynamics that requires specific and computationally expensive finite element models [47]. Nevertheless, even if the vibration signature depends on the specific topography and tribology of the defect, some studies relate the amplitude increase with the defect size and the shaft speed. According to [50], the increment in vibration is related to the defect size depending on the location and the defect size ratio ($D_{r}$), which is defined as follows:

(5) $$D_{r}=\frac{\theta_{d}}{\Delta \theta }=\frac{n_{b}\theta_{d}}{2\pi r}$$

where $\theta_{d}$ represents the defect size, *r* is the bearing radius corresponding to the race containing the defect arc, and Δθ is the circumferential space between two rolling elements. Figure 2 presents a graphical explanation of the defect ratio. For small defect ratio values (i.e., $D_{r}<1$) both inner and outer race defects present a linear relationship between the vibration rms value and the defect size. Thus, for increased speed and defect width, an increase in the vibration amplitude is expected.

### 2.2. Time–Frequency Envelope Spectrum

Vibration signals caused by localized defects in bearings are characterized as amplitude-modulated, with a high-frequency carrier signal being modulated by a lower-frequency component. Classic signal processing tools exploit the demodulation of the vibration signal to extract information from the quasi-periodic vibration pulses. The benchmark processing strategy for localized bearing fault diagnosis is the envelope spectrum analysis [19]. Vibration signals are first demodulated and then transformed in the frequency domain by utilizing the Fast Fourier Transform (FFT). Demodulation is performed using the Hilbert Transform (HT), which generates the complex analytic signal. Given a discrete, time-dependent signal x(t)=Acos(ωt), the corresponding analytic signal is defined as follows:

(6) $$HT\left[x(t)\right]=\hat{x}\left(t\right)=A\left[cos\left(\omega t\right)+j\;sin\left(\omega t\right)\right]$$

**Figure 3:**
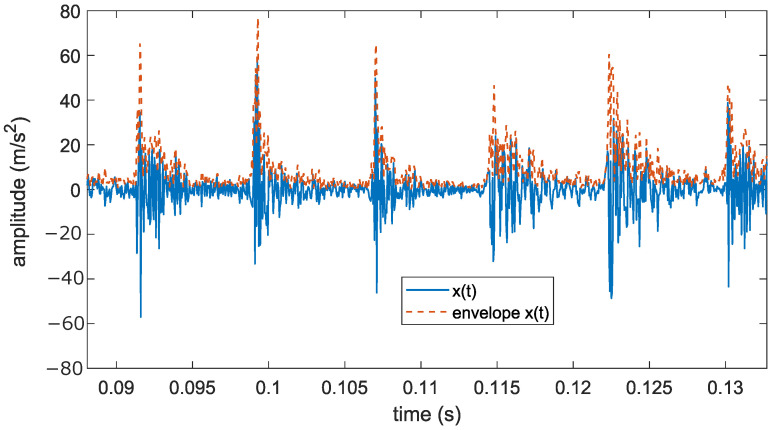
Bearing defect vibration signal x(t) and its envelope.

Note that the analytic signal is defined as a complex-valued function in which the imaginary part is 90° shifted with respect to the real function. The envelope of the signal x(t) is provided by the module of the analytical signal $\hat{x}\left(t\right)$. Figure 3 shows an example of a quasi-periodic bearing vibration signal and its envelope.

**Figure 4:**
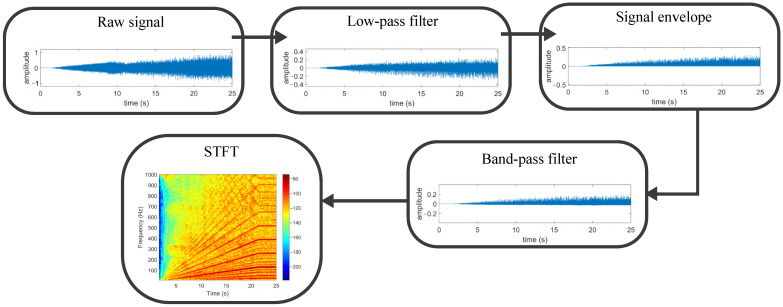
Signal processing pipeline graphic description with an inner race defect example.

Variable speed vibration signals are not periodic by nature. Thus, the direct application of the FFT to non-periodic signals does not provide physically meaningful results. Time–frequency transformations such as the STFT, DWT, or WVD overcome these limitations and provide qualitative and quantitative information regarding signal evolutions. The utilized signal processing method comprises several steps. First, a low-pass filter tuned at the defined upper frequency limit (i.e., 1000 Hz in the present study) is applied to the raw signal. Then, the envelope of the signal is obtained by utilizing the above-described HT. To effectively track significant bearing fault frequencies, the signal should be band-pass filtered before applying the STFT. This process primarily serves to filter out high-frequency components and to eliminate the DC offset from the envelope signal, which would otherwise dominate the signal if not removed. The band-pass filter lower cut-off frequency is defined at 5 Hz, while the upper limit is set at the highest frequency of interest. Finally, the signal is down-sampled to match the Nyquist frequency with the highest frequency of interest to further prevent aliasing phenomena. The present analysis utilizes the STFT to interpret the signature of the envelope spectrum during the machine start-up, which is a windowed version of the classic FFT. This tool is mainly selected due to its simplicity and low computational burden. The STFT is defined as follows:

(7) $$STFT\left[x(t)\right]=X\left(\tau ,f\right)=\int_{-\infty }^{\infty }x\left(t\right)\;w\left(t-\tau \right)e^{-j2\pi ft}dt$$

where τ is the window length, and the function w(t−τ) represents a window of length τ centered at instant *t*. In addition, a Hanning window is applied to each signal section to minimize spectral leakage, and a high window overlap level is applied to improve the time–frequency map resolution. Note that the STFT offers an adequate time–frequency resolution for the application at hand, but it presents a limited frequency resolution depending on the window length. Figure 4 depicts the signal processing pipeline used to achieve adequate time–frequency representation of the transient vibration signature.

## 3. Experiment Description

**Figure 5:**
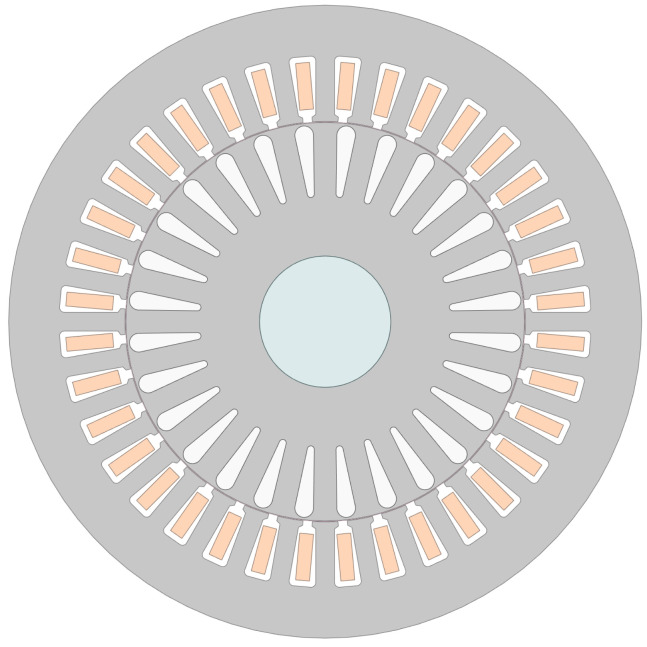
Induction motor specimen cross-section.

The proposed analysis is implemented by utilizing an induction motor test bench internally hosting the different bearings being tested. The utilized motor is a four-pole squirrel cage induction machine with 36 stator slots and 28 rotor bars. Figure 5 shows the machine cross-section, and Table 1 presents its main characteristics.

**Figure 6:**
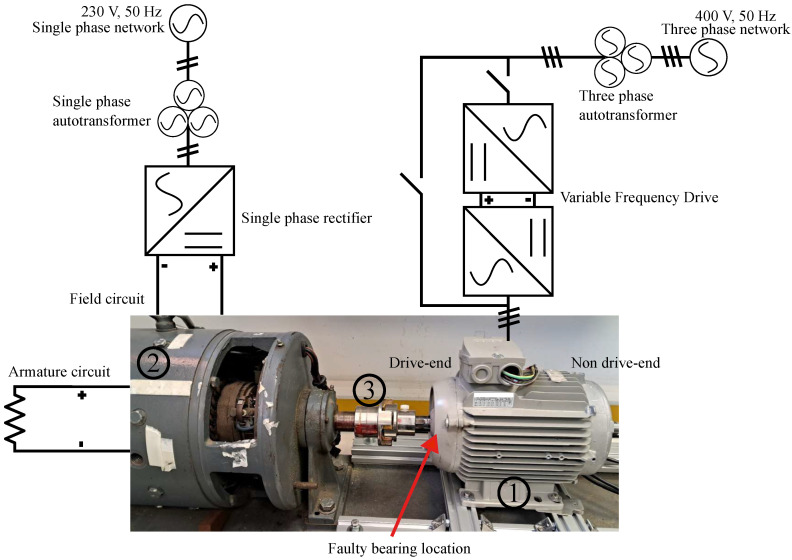
Test bench graphic description. (1) Induction machine including faulty bearing, (2) DC generator imposing constant resistant torque, (3) flexible coupling.

The bearings under test are internally allocated within the DE plate of the induction machine. This represents a realistic diagnosis scenario including the structural response of the motor and not only the bearing structural elements. The induction motor is coupled to a DC generator via flexible mechanical coupling that imposes a constant resistant torque. The load torque is manually controlled by utilizing a variable autotrasformer connected to the field winding. The armature winding of the DC generator is connected to a dissipation resistance. The induction machine is either directly supplied by a 50 Hz three-phase network or driven via a Variable Frequency Drive (VFD). The machine’s alignment is precisely adjusted using a commercial tool that quantifies misalignment, ensuring that the levels remain within standard limits. Figure 6 shows a schematic of the test bench.

**Figure 7:**
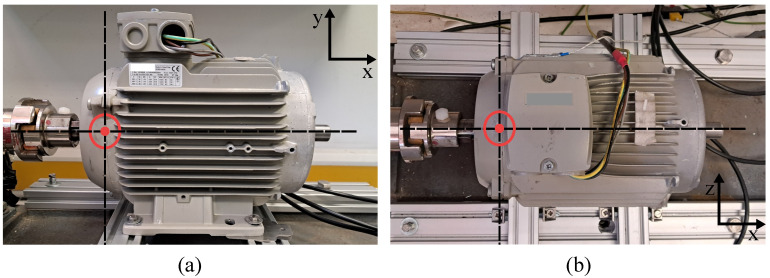
Accelerometer locus description. (**a**) Vertical xy-plane, (**b**) horizontal xz-plane.

The signal acquisition is performed by utilizing commercial piezoelectric unidirectional accelerometers (PCB352C33). These are placed in two orthogonal circumferential directions in the DE plane (i.e., located at 12 o’clock and 3 o’clock). In this way, the reliability of the measurement is improved by considering slightly different structural responses. The sensor is attached using a well-known adhesive polymer (UHU^®^ Patafix), which offers proper stability and attachment flexibility [51]. The sensors are connected to a signal conditioning unit, and the acquisition is performed using a wave recorder (Yokogawa DL350, Tokyo, Japan) at a sampling frequency of 20 kHz. Figure 7 shows the location description of the accelerometers in the induction machine specimen.

**Figure 8:**
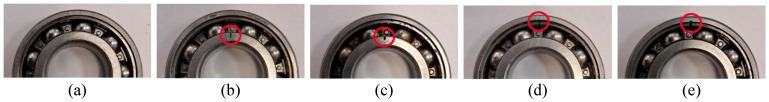
Bearing defect description. (**a**) Healthy, (**a**) 0.5 mm inner race defect, (**c**) 1 mm inner race defect, (**d**) 0.5 mm outer race defect, (**e**) 1 mm outer race defect.

This study includes different spherical rolling element bearings with different localized defects in the races. The defects are artificially induced via electric discharge machining in both the inner and outer races. Moreover, two different defect widths are implemented within the range of $D_{r}<1$, corresponding to widths of 0.5 and 1 mm, respectively. Figure 8 depicts the different bearings under test with the implemented race faults. Table 2 shows the main bearing dimensions according to Figure 1b, together with the expected fault signature in the frequency domain following Equations (1)–(4). The characteristic fault signature is provided in terms of a coefficient *k*, only including geometry characteristics, which multiplies the rotating frequency to determine the characteristic fault locus in the frequency domain. The faulty bearings are allocated within the DE end plate, as shown in Figure 6. Outer race defects are placed in the maximum radial load area at the 6 o’clock circumferential position.

**Figure 9:**
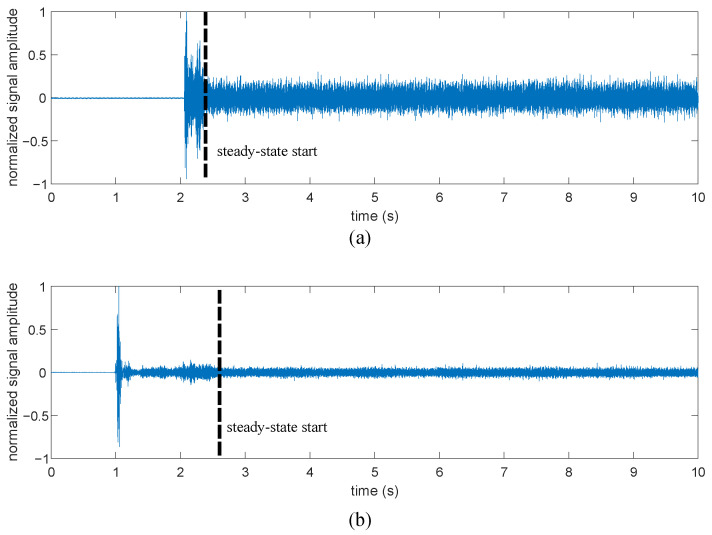
Line-fed induction machine startup vibration signal at 12 o’clock for (**a**) rated line-to-line voltage, (**b**) 50% rated line-to-line voltage.

The study of the bearing fault signature is performed during the machine’s transient start-up. This strongly varies depending on the electrical actuation of the induction machine. The present analysis examines two of the most commonly encountered actuation scenarios, specifically those involving VFD actuation and direct line-fed operation. The direct line-fed induction machine at the rated line-to-line voltage produces an extremely fast start due to the low inertia generally present in low-voltage machines. The abrupt start-up of the machine may mask the useful transient vibration response due to a high-amplitude initial shock. To mitigate this, a reduced voltage start-up at 50% of the rated line-to-line voltage is additionally implemented. In this way, the motor start-up is elongated and surpasses the duration of the acquired initial mechanical shock. Figure 9 exemplifies the differences between the rated and 50% line-to-line voltage start-ups at the rated steady-state slip and healthy bearing. The VFD-fed actuation is performed by utilizing a commercial inverter featuring an open-loop scalar control. The control system progressively varies the frequency and the supply voltage to keep a constant V/Hz ratio. This is performed at different variation rates, which are defined by a ramp length parameter. Thus, different transient start-up lengths are imposed to verify the behavior of faulty bearing components at different acceleration rates. The examined VFD-fed start-up ramps are defined with durations of 5 and 20 s, respectively.

The experimental campaign includes the vibration signal acquisition at different constant load points imposed by the DC generator. The machine load level is defined by the slip at steady-state (i.e., relative difference between synchronous and shaft rotating speeds). Note that the slip heavily swings from 1 to nearly 0 in the line-started case, while it is kept constant during the VFD-fed startup [52]. Five load points are defined, corresponding to the inherent slip at no-load and four points in steps of 25% of the rated slip (i.e., 1485 rpm, 1470 rpm, 1455 rpm, and 1440 rpm). Under conditions of reduced line-to-line voltage excitation, only four load points are defined, as the available torque scales with the square of the voltage. Additionally, no-load excitation already accounts for 25% of the rated slip. A total of 10 startups and steady-state signals of a 30 s duration are acquired per load point, startup type, and bearing fault topology.

## 4. Analysis of Results

This section presents the acquired results both in steady-state and different transient startup signals. Moreover, the results are compared with an open-source dataset, where faulty bearing vibration signals are directly acquired in the bearing surroundings during a VFD-fed startup.

### 4.1. Steady-State Analysis

The steady-state analysis at the rated slip of the vibration envelope spectrum elucidates the different fault signatures in the acquired signals. The spectrum is obtained by enveloping the vibration signal and further transforming it in the frequency domain though the well-known FFT. A Hanning window is implemented to reduce spectral leakage.

**Figure 10:**
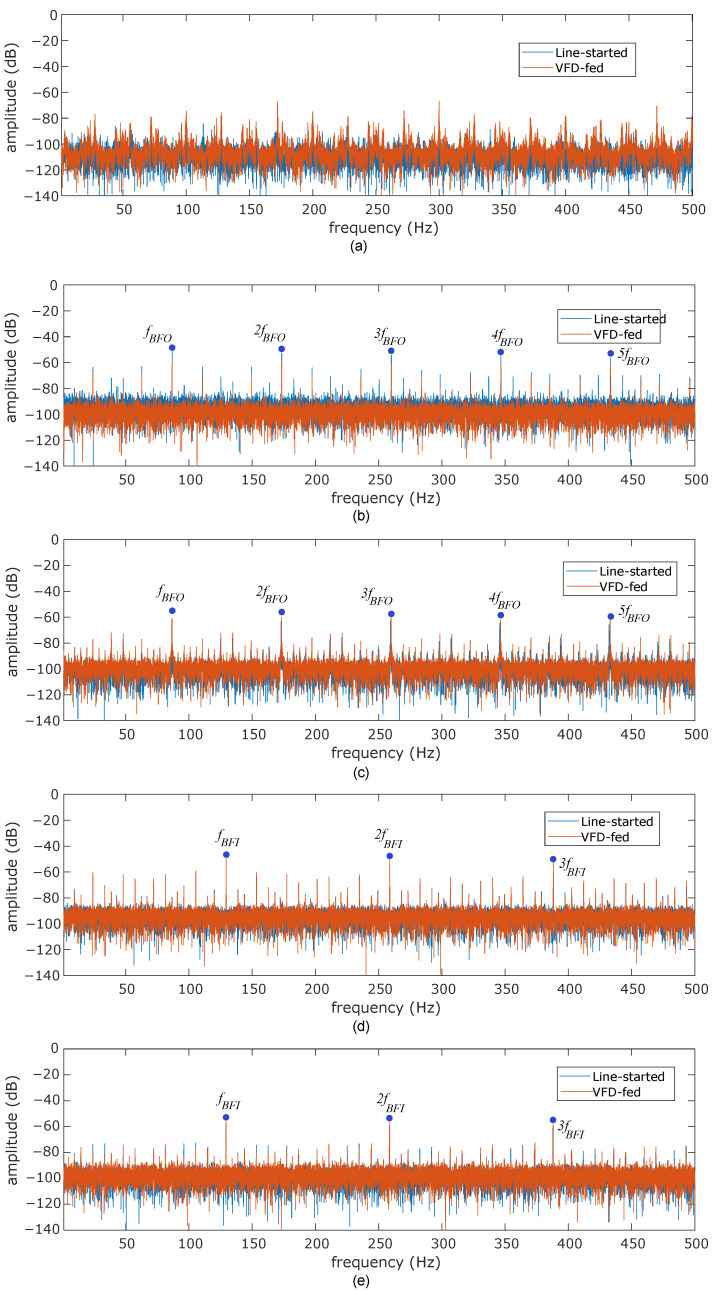
Vibration envelope spectrum analysis acquired at 12 o’clock position at rated slip, (**a**) healthy, (**b**) 0.5 mm outer race defect, (**c**) 1 mm outer race defect, (**d**) 0.5 mm inner race defect, (**e**) 1 mm inner race defect.

**Figure 11:**
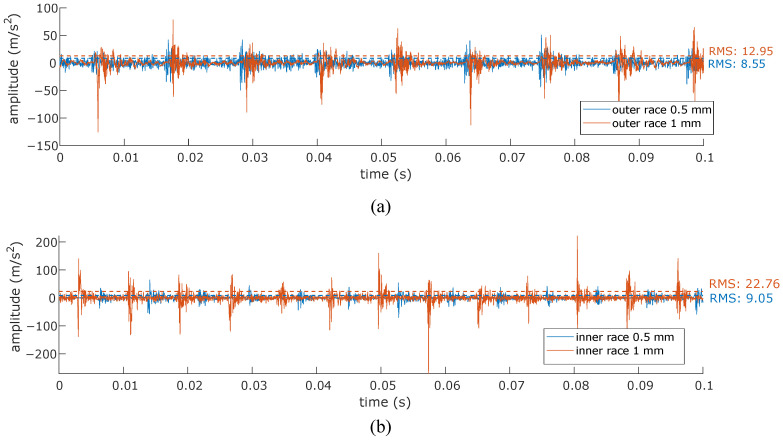
Vibration amplitude comparison among two defect widths. Signals acquired at 12 o’clock at rated slip. (**a**) Outer race defects, (**b**) inner race defects.

Figure 10 shows the envelope spectrum for different bearing specimens, including healthy, inner race, and outer race defects. The spectrum includes signals acquired during line starting at rated line-to-line voltage and VFD-fed excitation. The displayed signals are acquired at the 12 o’clock circumferential position at the rated slip (i.e., 1440 rpm shaft rotation). An immediate observation is the clear identification of the characteristic fault frequencies corresponding to inner and outer race-localized defects in both the line-started and VFD-fed cases. The envelope spectrum in healthy conditions does not show relevant information regarding bearing defects and only highlights the increased noise for VFD-acquired signals. Note that the envelope spectrum components observed in the healthy case corresponding to Figure 10a are not present in the faulty cases. This fact emphasizes the absence of significant electromagnetic vibration components influencing the bearing fault detection within the frequency range of interest when demodulation tools are utilized. Table 3 shows quantitative data regarding the amplitude and frequency location of characteristic faulty components for both defect sizes and actuation modes. A first conclusion drawn from the steady-state analysis is that the fault frequencies are independent of the actuation mode, as evidenced by the nearly identical amplitude values observed for both the line-started and VFD-fed modes. The light changes observed in the frequency location are derived from the manual tuning of the shaft speed by utilizing a manual tachometer and from the expected rolling element slippage. The amplitude comparison between 0.5 mm and 1 mm defects shows a slight decrease for the higher defect level. This fact contrasts with the expected rms increase in the vibration signal for higher defect sizes [50]. Nevertheless, the amplitude of the mechanical shocks follows the expected rms vibration increase, as shown in Figure 11. This increase is better observed for inner race defects in the present study. In addition, rotating frequency-modulated components around fault characteristic frequencies (i.e., $kf_{BPI}\pm nf_{r}\;\;\forall \;\;k=1,2,3,\cdots \;\;n=1,2,3,\cdots$; $kf_{BPO}\pm nf_{r}\;\;\forall \;\;k=1,2,3,\cdots \;\;n=1,2,3,\cdots$) are more prominent for reduced defect sizes.

### 4.2. Transient Analysis

**Figure 12:**
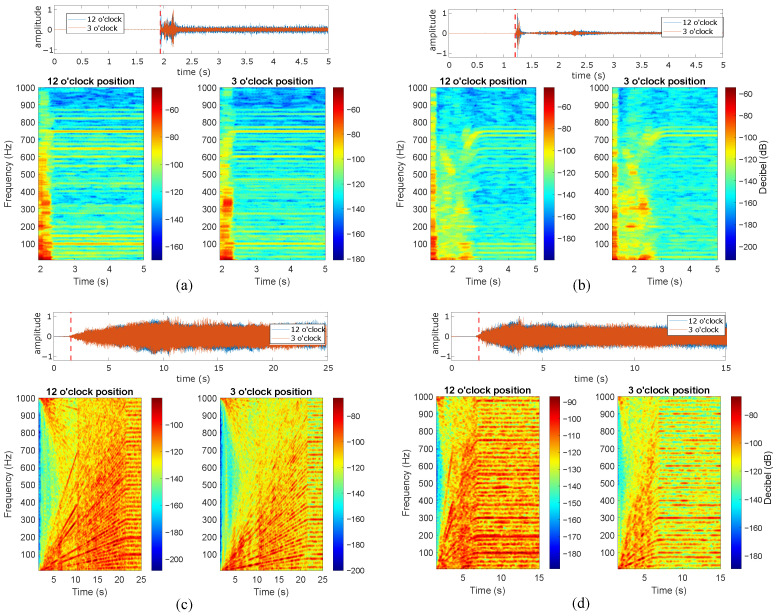
Healthy bearing at rated slip, (**a**) line-started 100% rated voltage, (**b**) line-started 50% rated voltage, (**c**) VFD-fed 20 s ramp, (**d**) VFD-fed 5 s ramp.

**Figure 13:**
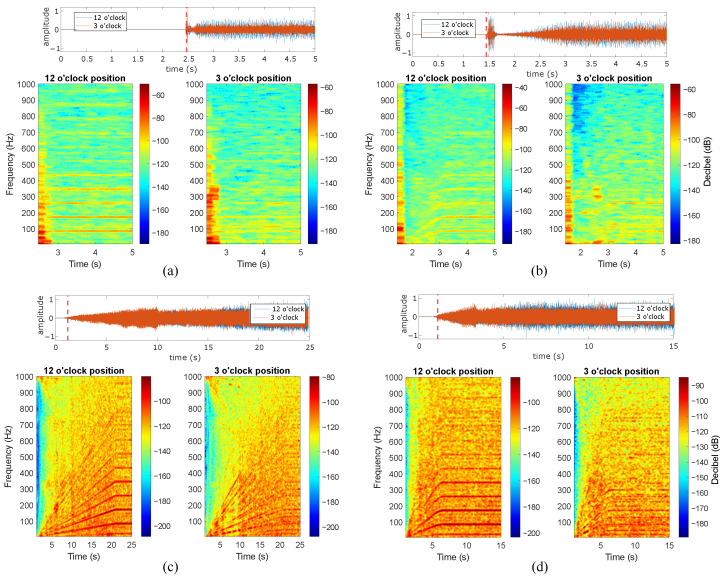
Outer race 0.5 mm defect at rated slip, (**a**) line-started 100% rated voltage, (**b**) line-started 50% rated voltage, (**c**) VFD-fed 20 s ramp, (**d**) VFD-fed 5 s ramp.

**Figure 14:**
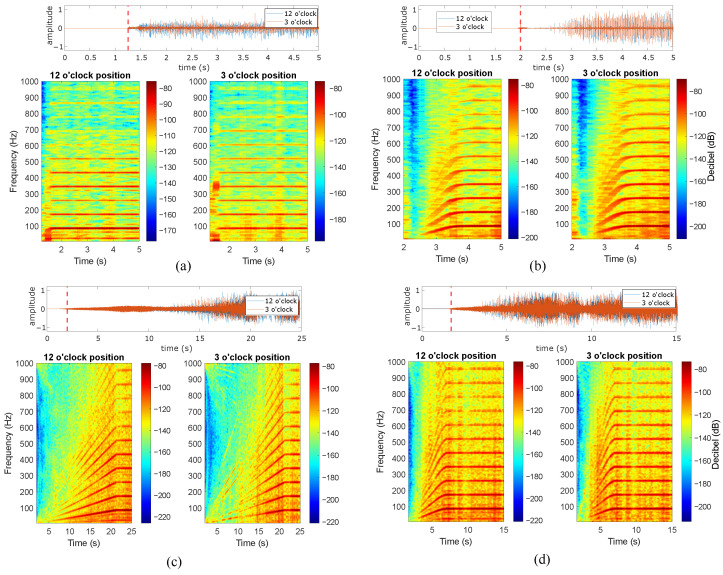
Outer race 1 mm defect at rated slip, (**a**) line-started 100% rated voltage, (**b**) line-started 50% rated voltage, (**c**) VFD-fed 20 s ramp, (**d**) VFD-fed 5 s ramp.

**Figure 15:**
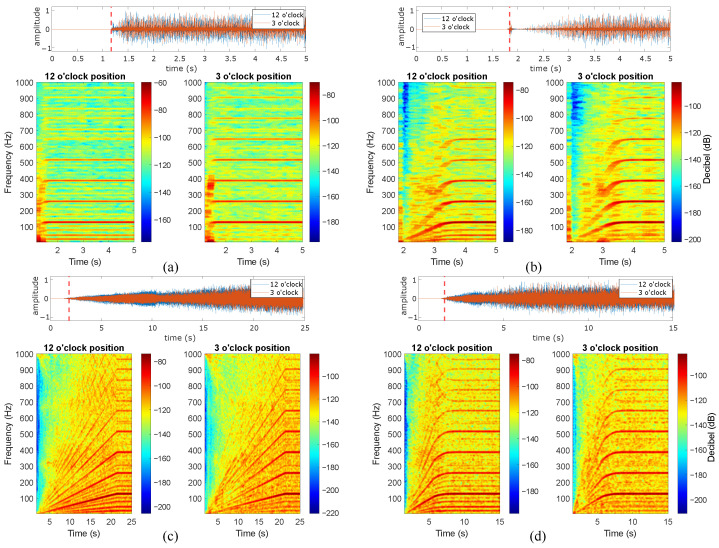
Inner race 0.5 mm defect at rated slip, (**a**) line-started 100% rated voltage, (**b**) line-started 50% rated voltage, (**c**) VFD-fed 20 s ramp, (**d**) VFD-fed 5 s ramp.

**Figure 16:**
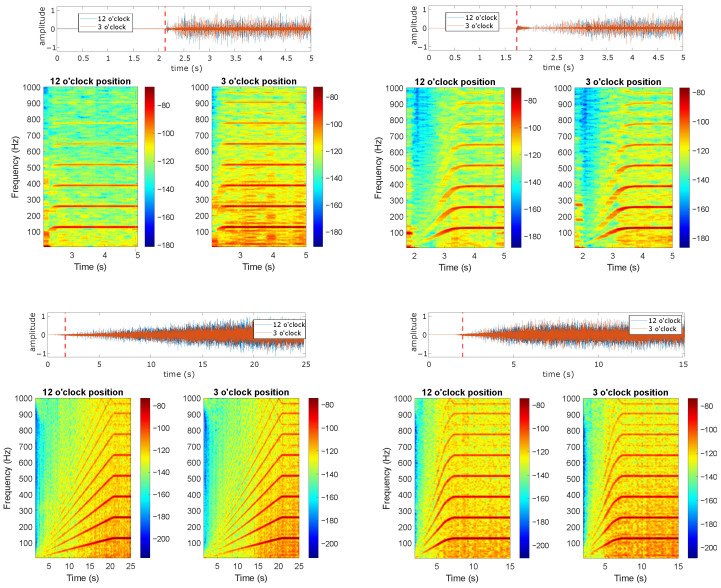
Inner race 1 mm defect at rated slip, (**a**) line-started 100% rated voltage, (**b**) line-started 50% rated voltage, (**c**) VFD-fed 20 s ramp, (**d**) VFD-fed 5 s ramp.

The present subsection describes the analysis of the induction motor vibration signals during the studied start-up excitation modes at the rated slip. Four distinct start-up modes were implemented, including line-started at 100% and 50% of the rated line-to-line voltage, along with VFD-fed modes featuring ramp-up times of 20 s and 5 s. Figure 12, Figure 13, Figure 14, Figure 15 and Figure 16 show the results of the start-up analysis by utilizing the demodulated STFT together with the time-domain vibration signals at both the 12 o’clock and 3 o’clock circumferential positions. The dashed red line marks the start point of the time–frequency transformation, which is applied to exclude the processing of blank signal intervals. The STFT time window (i.e., w(t−τ)) is kept at 0.3 s across the different start-up scenarios to allow for the quantitative analysis of time–frequency amplitudes.

Figure 12 shows the vibration signature when a brand new healthy bearing is located in the end plate. The effects of the VFD actuation are clearly identified by observing the time–domain transient signals. The VFD-induced harmonic content in the vibration signals is mainly located at higher frequencies, as evidenced by the steady-state comparison in Figure 10 and Table 3 even if the noise floor in the frequency range of interest is clearly increased. By observing the line-started signals, the overall vibration amplitude is lower for the reduced line-to-line voltage case. This observation is aligned with the expected electromagnetic vibration for reduced excitation levels, even if the shaft rotates at the rated speed. Moreover, in the case of the healthy bearing in line-started conditions, a high-amplitude starting shock is observed for both voltage levels. Sensors located at 12 o’clock and 3 o’clock provide similar information, with some amplitude differences, mainly due to the structural transfer function between acting forces and vibration acquisition points.

Figure 13 and Figure 14 show the transient vibration analysis of the two studied bearings containing outer race defects. The outer race defect signature $kf_{BPO}\;\forall \;k=1,2,3,\cdots$ is clearly observed in all cases. However, the outer-race 0.5 mm defect provides less-evident signatures in both the line-started and VFD-fed excitation modes. The main reason for the signature masking in the case of the line-started 0.5 mm defect is the presence of a high-amplitude initial shock. In addition, the signature of this defect is not evident in the case of the 3 o’clock position, even in the case of VFD-fed excitation. For the 1 mm outer race defect case, the fault signature during transient evolution is much more observable. In this case, no initial shock is observed in the case of line-started excitation, which contributes to the clear identification of $f_{BPO}$ frequencies. The fault signature is not evident at low rotating speeds. This is clearly observed in the transient evolutions of 50% line-started and VFD-fed excitation modes, even in the time domain signals. Moreover, the signature becomes observable at both the 12 o’clock and 3 o’clock positions, with only slight differences observed during long ramp excitation.

Figure 15 and Figure 16 present the transient vibration analysis for the two bearings with inner race defects under study. Both 0.5 mm and 1 mm defects clearly provide observable fault signatures at the characteristic frequencies $kf_{BPI}\;\forall \;k=1,2,3,\cdots$. Moreover, no initial shock masking is observed in any inner race case, which allows for the clear identification of characteristic frequencies in the line-started cases. All signatures are clearly evidenced in both the acquisition circumferential positions. In addition, the characteristic components are observed independently of the rotation speed for the VFD case. Under faster acceleration at 50% of the rated line-to-line voltage, the initial portion of the signal shows no contact shocks, making the characteristic frequencies unobservable at low speeds.

The mechanical impulses induced by race defects dominate the signal envelope spectrogram during the start-up for all types of actuation. The inner race is the most observable race defect for both defect widths, while the outer race 0.5 mm defect provides a less-dominant signature, even if the characteristic frequencies are clearly observed in the envelope spectrogram. The acquisition position in the circumferential direction does not heavily affect the bearing signature identification, which is only influenced under weaker excitation levels (e.g., outer race 0.5 mm defect or healthy specimen). On the other hand, the presence of initial mechanical shocks heavily hinders bearing signature identification during machine start-up. Another observation is the low levels of the characteristic fault frequencies for low speeds during the initial start. This initial attenuation is better observed for 1 mm defects in outer and inner races, while smaller defect widths are evident even for initial rotation phases.

### 4.3. Effects of Load on the Characteristic Defect Signature

**Figure 17:**
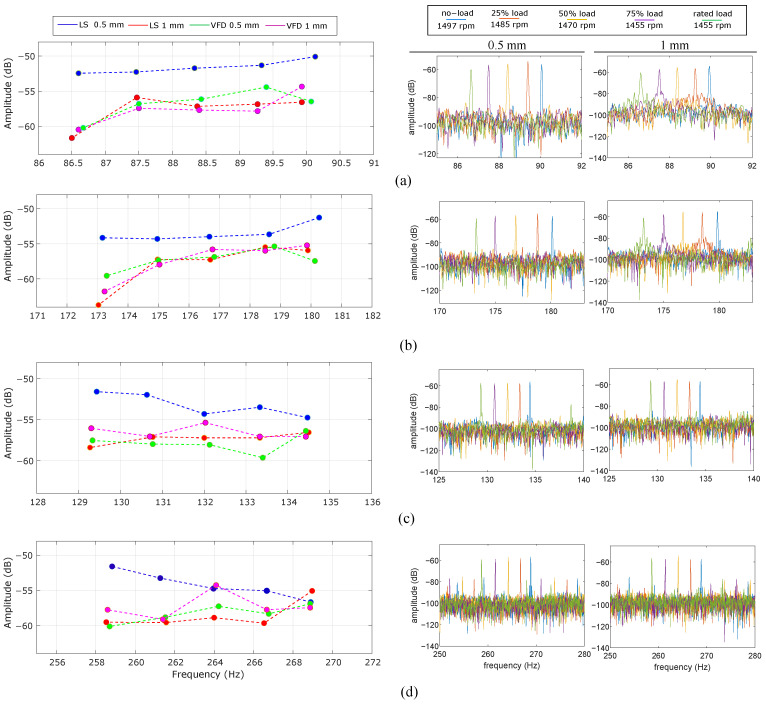
Load dependency steady-state analysis. (**a**) $f_{BFO}$, (**b**) $2f_{BFO}$, (**c**) $f_{BFI}$, (**d**) $2f_{BFI}$.

The present subsection analyzes the effects of machine load on the analytically estimated characteristic frequencies for both steady-state and transient conditions. This analysis is performed to verify the presence of the bearing fault components under different electromagnetic and speed conditions. For increased load levels, the slip slightly increases and the induction motor absorbs higher phase current levels, which may influence the level of electromagnetic vibration. The load variation analysis is performed for both line-started and VFD-fed cases as well as for different bearing defect widths. Figure 17 shows the steady-state analysis of the first two faulty components (i.e., $f_{BFO}$, $2f_{BFO}$, $f_{BFI}$, $2f_{BFI}$) for different load points. The left side of the figure shows the amplitude trends for different load points, while the right side shows the frequency locus of the different fault signatures for different load points. These right-side graphs are plotted for the VFD-fed case.

The faulty bearing components are identified independently of the load. A notable observation is the shift in frequency locus as a function of the load point. This is consistent with the expected variation in slip due to load changes in induction machines. Consequently, the characteristic faulty frequencies dynamically change with machine speed and the applied load. Nevertheless, the fault frequency does not exhibit significant variations across different load points, remaining within a maximum relative 4% deviation. By observing the amplitude trends of the outer race fault frequencies (i.e., Figure 17a,b), the amplitude generally decreases for increased load levels and lower speeds. On the other hand, the inner race signature does not exhibit a clear amplitude trend. Additionally, the excitation mode does not significantly affect the amplitude of the characteristic frequency at different load points. The data presented in Figure 17 further support the findings of Figure 10 and Table 3, where the defect width is not identified as a significant parameter regarding the characteristic frequency amplitudes. The data corresponding to the line-started 0.5 mm defect demonstrate an increased noise floor, causing the amplitudes to be at a slightly higher level. Note that the absolute amplitude values may show slight inaccuracies due to several phenomena such as parasitic load oscillations or bearing slippage, even if energy leakage is minimized by utilizing signal windowing.

**Figure 18:**
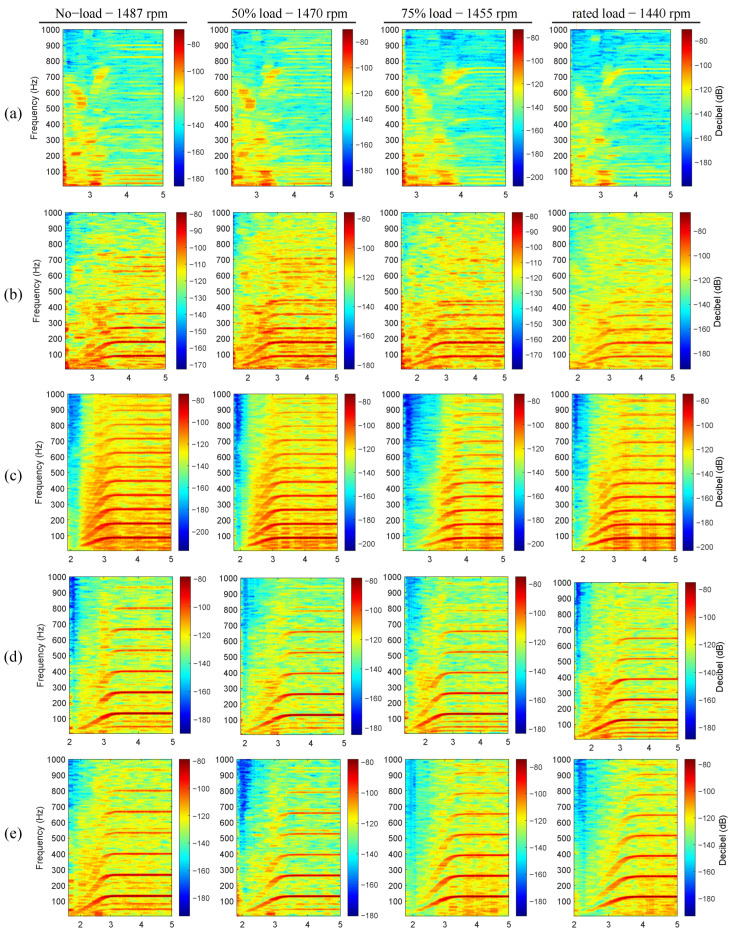
Load variation analysis during the line-started excitation mode at 50% rated line-to-line voltage. Vibration signals acquired at 12 o’clock. (**a**) Healthy bearing, (**b**) outer race 0.5 mm defect, (**c**) outer race 1 mm defect, (**d**) inner race 0.5 mm defect, (**e**) inner race 1 mm defect.

**Figure 19:**
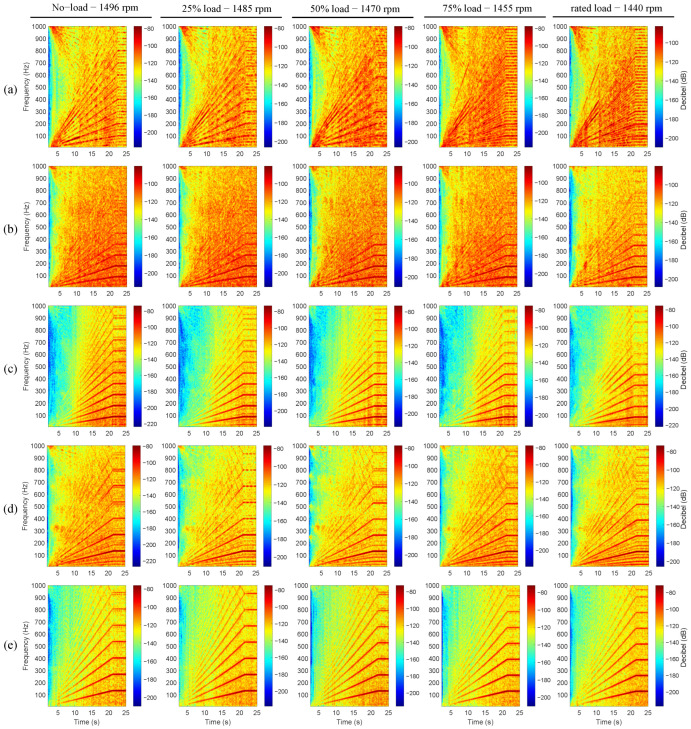
Load variation analysis during the VFD-fed excitation mode with 20 s ramp duration. (**a**) Healthy, (**b**) outer race 0.5 mm defect, vibration signals acquired at 12 o’clock, (**c**) outer race 1 mm defect, (**d**) inner race 0.5 mm defect, (**e**) inner race 1 mm defect.

Figure 18 and Figure 19 show different start-up transient evolutions for different imposed resistant torques. The vibration signals were acquired at 12 o’clock and were plot after the initial shock appearance to better highlight variations across faulty components. Figure 18 shows the results of the study for the line-started actuation mode at 50% of the rated line-to-line voltage. This excitation mode is selected to avoid the fast slip swing observed at 100% of the rated line-to-line voltage. The analysis of the line-started results evidences the presence of bearing faulty components across different resistant torque levels and speeds. Both multiples of $f_{BFO}$ and $f_{BFI}$ are dominant for all load levels and defect widths during the transient start-up and once steady-state conditions are reached. The only effect of the load level is a minimal variation in the start-up duration, which is nearly imperceptible when analyzing the time–frequency maps.

Figure 19 shows the VFD-fed load analysis during a 20 s ramp start-up. This ramp is selected due to the increased transient signal section when compared with the 5 s start. The figure shows the dominance of the faulty characteristic frequencies across the studied load points. Note that even the amplitude levels are within the same ranges across all of the different loads and defect cases. The analysis presented in both Figure 18 and Figure 19 indicates the load independence of the characteristic fault signature for all defect widths, load levels, and start-up modes and durations.

### 4.4. Comparison with HUST Dataset

**Figure 20:**
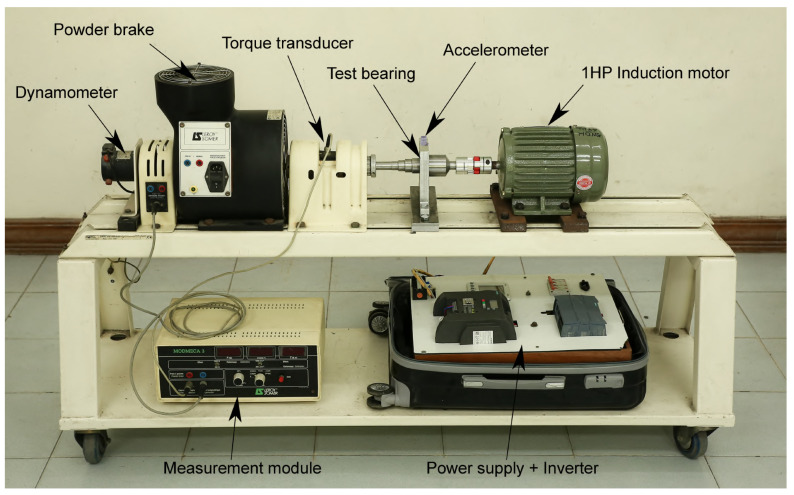
HUST dataset experimental test bench description [53].

The present subsection compares the acquired transient signature with a recently published open-source dataset. This is performed to further elucidate the effects of the mechanical transfer path and to allow for a deeper analysis of the results. The HUST dataset [53] includes vibration data acquired on a dedicated bearing module, thus avoiding vibrations of electromagnetic and mechanical origin from the machine itself. The dataset contains data from different faulty bearings at different loads imposed by a controlled powder brake. The bearings are damaged using electric discharge machining with a defect with of 0.2 mm. The same accelerometer (i.e., PCB325C33) was utilized to acquire the vibration data, which contains VFD-fed start-ups of a 5 s duration. Figure 20 shows the HUST dataset experimental set-up.

**Figure 21:**
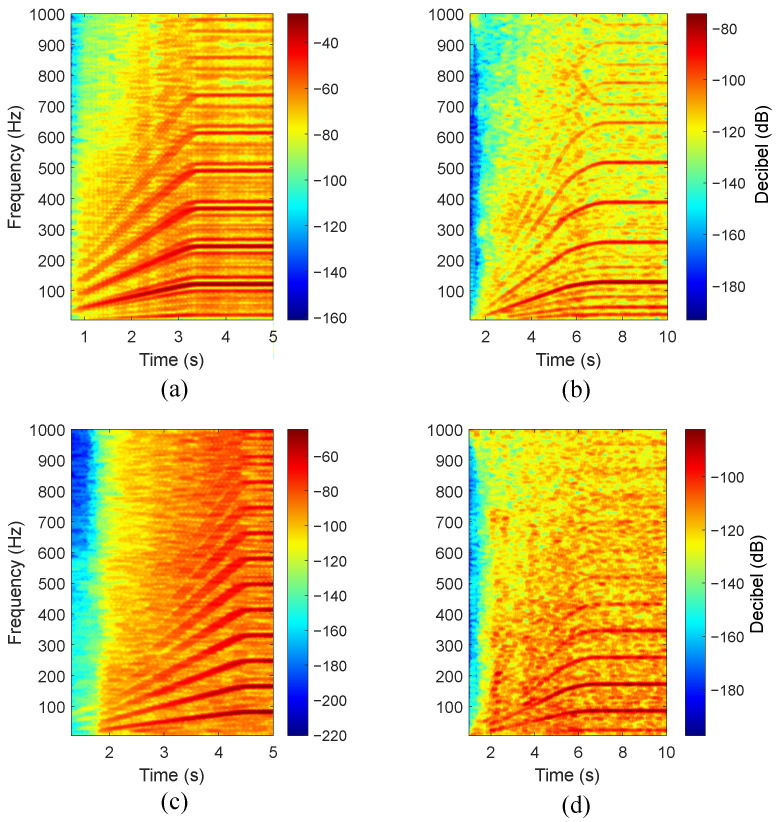
VFD-fed start-ups for inner and outer race defects, (**a**) HUST dataset inner race defect, (**b**) custom dataset inner race defect, (**c**) HUST dataset outer race defect, (**d**) custom dataset outer race defect.

The qualitative comparison between datasets was performed under the closest possible conditions in terms of bearing dimensions and start-up duration. Thus, the inner and outer race defect data corresponding to the HUST bearing labelled as 6205 are used, since they possess the same number of rolling elements and similar inner, outer, and ball diameters to the bearing studied in the custom dataset. Moreover, the maximum load point is selected (i.e., 800 W load) in the case of the HUST dataset, providing a shaft speed of approximately 1370 rpm. The custom data are shown at the rated induction motor load point featuring a 1440 rpm shaft speed. Figure 21 shows the different time–frequency maps for the selected signal comparison.

Figure 21 clearly shows the expected characteristic defect signatures for all the types of studied bearing defects during both transient start-up and steady-state sections. An immediate observation is the presence of increased rotating frequency modulations in Figure 21a when compared with the signals acquired in the motor housing. This is evidenced by the components $kf_{BPI}\pm f_{r}\;\forall \;k=1,2,3,\cdots$, which are not observed in Figure 21b. The comparison between outer race defects proves the increased difficulty to discern characteristic faulty components when compared to the inner race case. Moreover, Figure 21c shows very small amplitudes in the beginning of the start-up evolution, which is in accordance with the signals shown in Figure 14 and Figure 18. Overall, each set-up exhibits distinct characteristics; however, the studied defects are clearly identifiable during both the transient start-up and steady-state phases. Nevertheless, the comparison demonstrates that the mechanical transfer path plays a key role in elucidating the characteristic signature and its rotating frequency modulations. The significance of the mechanical transfer path limits the generalizability across different induction machine specimens with varying structural characteristics (i.e., mass, stiffness, and damping), and power ratings.

## 5. Discussion

The analysis of low-frequency bearing defect characteristic frequencies performed in this work supports the detection feasibility of incipient localized bearing defects across different operating regimes and actuation modes in induction motors. These low-frequency components are identified using a simple and linear time–frequency transformation of the signal envelope. This indicates that the utilization of complex signal processing tools may not represent an efficient practice when a low-frequency characteristic fault signature is targeted. The utilized time–frequency transformation, which contains physical information about the fault, may be of interest for physics-informed data-driven approaches, which typically require the preprocessing of large volumes of data. The vibration envelope spectrum analysis of low-frequency characteristic fault components cannot properly discriminate between defect widths. Different defect widths in the range of $D_{r}<1$ provide similar amplitudes across different load points and excitation modes. This is extended to time–frequency analysis of variable speed vibration signals. Finally, the observability of the characteristic frequency across different machine regimes aligns with signals from datasets obtained under slightly different mechanical conditions, as discussed in Section 4.4.

This study presents several limitations, such as the artificial implementation of defects, which may slightly differ from incipient naturally induced cracks. The presence and amplitude of outer race shocks heavily depends on the defect circumferential position, which is kept at the loaded area (i.e., 6 o’clock) for all studied faults in the present analysis. Outer race defects are only studied when they are located in the loaded zone. Moreover, the induction machine specimen is disassembled to change the bearing under test. This may slightly affect the mechanical features of the test bench, even if attention is paid to keep the assembly methodology as standard as possible. Finally, spurious mechanical shocks of a different nature may mask the bearing defect if these are included in the analyzed signal sections.

## 6. Conclusions

The present paper introduced an experimental study regarding localized bearing fault detection across different operating regimes in induction motors. First, this work introduced the relevant theoretical background to properly interpret the analysis and to describe the utilized time–frequency representation tool. Next, the experimental procedure was described in detail, including the induction motor test bench, the vibration signal acquisition system, the bearing fault implementation, and the presence of spurious mechanical shocks that may mask the characteristic fault signature. The analysis of the experimental results is presented in several steps. First, a classic steady-state analysis at the rated slip was conducted. Second, a broad start-up transient exploration at the rated slip, including different excitation modes, bearing defect widths, and acquisition circumferential positions, was performed. Third, the effects of load and thus light changes in shaft speed and imposed constant resistant torque were studied. To conclude the analysis, the acquired data were compared with the corresponding data from an existing open-source dataset. The utilized time–frequency envelope spectrum was used to identify the characteristic bearing fault signature independently of the start-up mode, defect width, and load point. This allows us to distinguish between defect locations during start-up in an efficient and straightforward manner across many operating scenarios. The mechanical transfer path of the induction machine influences the bearing defect signature transmission, as demonstrated by the comparison of signals acquired near the bearing with those measured at the motor housing.

Future research steps include the study under different lubrication states and outer race defects locations in the circumferential position. The applicability of the detection technique should be validated for machines with different masses, power ratings, and features in order to further generalize it. This may include a detailed structural analysis under different bearing defects and closely monitoring the test bench mechanical features. Moreover, the relation between defect width and rotating frequency modulating harmonics in the envelope spectrum should be explored in detail for both inner and outer race defects.

## Figures and Tables

**Table 1:** Induction motor characteristics.

Number of poles	4
Rated power	1.1 kW
Rated speed	1440 rpm
Power factor	0.78
Rated voltage	230/400 V
Number of rotor bars	28
Number of stator slots	36

**Table 2:** Bearing dimensions with $n_{b}=9$ and characteristic frequency coefficients *k* assuming θ=0.

$d_{in}$ [mm]	$d_{out}$ [mm]	$b_{d}$ [mm]	$d_{p}$ [mm]
25	52.00	7.94	38.5
***BFO***	***BFI***	***BSF***	***CF***
3.59	5.41	2.37	0.40

**Table 3:** Characteristic frequency location and amplitudes for different faulty bearings.

	Line-Started		VFD-Fed	
	Frequency [Hz]	Amplitude [dB]	Frequency [Hz]	Amplitude [dB]
	Outer race, 0.5 mm			
$f_{BFO}$	86.67	−51.34	86.67	−60.21
$2f_{BFO}$	173.31	−52.82	173.31	−59.53
$3f_{BFO}$	260	−54.58	259.96	−61.24
	Outer race, 1 mm			
$f_{BFO}$	86.44	−62.4	86.6	−60.47
$2f_{BFO}$	172.9	−63.32	173.23	−60.32
$3f_{BFO}$	259.32	−63.33	259.83	−60.32
	Inner race, 0.5 mm			
$f_{BFI}$	129.37	−49.95	129.4	−49.81
$2f_{BFI}$	258.74	−51.12	258.8	−51.63
$3f_{BFI}$	388.1	−52.88	388.17	−52.98
	Inner race, 1 mm			
$f_{BFI}$	129.27	−58.39	129.33	−56.15
$2f_{BFI}$	258.53	−59.52	258.67	−56.63
$3f_{BFI}$	387.8	−61.19	388	−57.96

## Data Availability

The HUST dataset analyzed in this study is openly available from Mendeley Data at 10.1186/s13104-023-06400-4 [53].

## Abbreviations

The following abbreviations are used in this manuscript:

STFT	Short-Time Fourier Transform
CWRU	Case Western Reserve University
DWT	Discrete Wavelet Transform
EMD	Empirical Mode Decomposition
CWT	Continuous Wavelet Transform
WVD	Wigner–Ville Distribution
MUSIC	Multiple Signal Classification
DE	Drive End
FFT	Fast Fourier Transform
HT	Hilbert Transform
VFD	Variable Frequency Drive
